# Mechanical Ventilation‐Associated Changes in Hippocampal Electroencephalogram: A Cross‐Species Study in Humans and Rats

**DOI:** 10.1002/cns.71012

**Published:** 2026-07-15

**Authors:** Xiang Qi, Xiaoyu Ou, Jingyi Li, Meizhizi Zhang, Fenqin Xue, Hua Wei, Wenting Su, Jing Wang, Yongxing Sun, Jian Zhou, Baoguo Wang, Zhonghua Shi

**Affiliations:** ^1^ Department of Neurosurgery, Department of Anesthesiology, Sanbo Brain Hospital Capital Medical University Beijing China; ^2^ Laboratory for Clinical Medicine Capital Medical University Beijing China; ^3^ Beijing Key Laboratory of Traffic Data Mining and Embodied Intelligence, School of Computer Science and Technology Beijing Jiaotong University Beijing China; ^4^ Department of Neurosurgery, Department of Intensive Care Medicine, Sanbo Brain Hospital Capital Medical University Beijing China; ^5^ Laboratory of in Vivo Electrophysiology Core Facility Center of Capital Medical University Beijing China; ^6^ Beijing Institute of Brain Disorders, Laboratory of Brain Disorders, Ministry of Science and Technology, Collaborative Innovation Center for Brain Disorders Capital Medical University Beijing China; ^7^ Epilepsy Center, Sanbo Brain Hospital Capital Medical University Beijing China; ^8^ Department of Neurosurgery, Sanbo Brain Hospital Capital Medical University Beijing China; ^9^ Department of Physiology Amsterdam University Medical Centre Amsterdam the Netherlands

**Keywords:** cross‐species comparison, human, intracranial electroencephalogram, mechanical ventilation, rat, ventilator‐associated brain injury

## Abstract

**Aims:**

To identify hippocampal intracranial electroencephalography (iEEG) signatures associated with mechanical ventilation (MV) in humans and rats and assess their translational relevance.

**Methods:**

Hippocampal iEEG was recorded in humans (*n* = 9) and rats (*n* = 12; MV + propofol vs. propofol‐only, *n* = 6 per group). Power spectral density (PSD), spectral exponent (SE), and trajectory entropy (TE) were quantified to characterize MV‐related neural changes.

**Results:**

MV was associated with consistent directional hippocampal iEEG changes across species, with increased PSD, SE, and reduced TE. In humans, PSD increases were mainly posterior (delta‐alpha; *p* < 0.05), while in rats they spanned dorsal theta‐gamma (*p* < 0.05). SE increased in humans (38.5%, *p* < 0.001) and rats (26.6%, *p* < 0.01), while TE decreased in humans (11.2%, *p* < 0.001) and rats (6.9%, *p* = 0.001). Cross‐species differences were greater in the posterior/dorsal hippocampus (PSD: d = −0.924 [−1.521 to −0.328]; SE: d = 0.944 [0.346 to 1.542]) than in anterior/ventral regions. In rats, MV increased low‐frequency power and SE and reduced dorsal TE under propofol.

**Conclusions:**

MV is associated with comparable hippocampal iEEG changes across species, supporting the rat model for MV‐related neural dynamics. Nevertheless, the findings are EEG‐restricted and lack general translational validity for brain injury.

**Clinical Trail Registration:**

ClinicalTrails.gov (NCT06480162; Zhonghua Shi; May 8, 2024)

## Graphical abstract

By comparing rats with humans across species, we found that MV exhibited similarities in the iEEG changes of the hippocampus in different species, and these effects occurred independently of propofol anesthesia, providing support for the rat model of the neural dynamic mechanism related to MV.

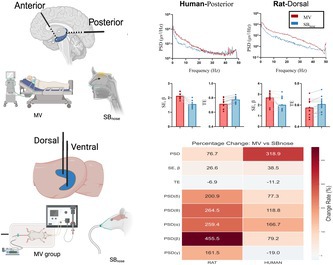

## 1 Introduction

Mechanical ventilation (MV) provides essential respiratory support during general anesthesia and for patients with acute respiratory failure. While life‐saving, emerging evidence suggests that invasive MV may contribute to brain injury, reflected by neuroinflammation, neuronal apoptosis, and cellular stress responses in brain tissues [1, 2, 3]. Clinically, MV is associated with neurocognitive complications, including acute delirium [4] and long‐term cognitive decline [5, 6], which link to increased ICU length of stay, prolonged hospitalization, and higher morbidity and mortality [7, 8, 9]. These observations have given rise to the concept of ventilator‐associated brain injury (VABI), a framework describing the potential adverse effects of MV on the central nervous system [1]. However, the early functional neural alterations potentially preceding overt structural or molecular injury remain incompletely characterized.

Among brain structures implicated in VABI, the hippocampus appears particularly vulnerable due to its sensitivity to hypoxemia, systemic inflammation, and sedatives [10, 11, 12, 13]. Beyond memory and spatial navigation [14, 15], the hippocampus also modulates arousal, sensorimotor integration, and autonomic regulation [12, 16]. Given its functional importance and susceptibility, the hippocampus has been proposed as a potential target of MV‐associated effects [1, 16], yet its electrical activity during MV remains poorly understood, which may provide early indicators of cognitive impairment [17]. Characterizing these electrophysiological dynamics may therefore provide insight into neural vulnerability and early functional perturbations associated with MV, even in the absence of direct structural injury markers.

In patients with drug‐resistant epilepsy, intracranial electroencephalogram (iEEG) provides valuable access to in vivo hippocampal activity [18, 19], offering a unique window to study MV‐related neural dynamics under clinical conditions. However, these recordings are constrained by the short‐term MV exposure, relatively preserved respiratory mechanics, and epilepsy‐related network alteration, limiting their applicability to prolonged MV paradigms. Rodent models, by contrast, enable precise control of MV parameters and longer‐duration iEEG acquisition, facilitating mechanistic exploration of MV‐associated neural signatures. However, the translational relevance of the preclinical findings in rats to human brain physiology during MV remains untested.

Therefore, the present study focuses on a cross‐species comparison of the dynamic hippocampus iEEG changes in humans and rats following short‐term MV. We hypothesized that MV is associated with temporally and spectrally similar hippocampal activity patterns across species, reflecting shared MV‐related neural dynamics. These findings may provide a framework for future mechanistic investigations of MV‐associated neural modulation and support the development of translational approaches to monitor and understand MV‐associated effects on brain activity.

## 2 Methods

### 2.1 Cross‐Species Comparative Study Design

Human data were obtained from a previously approved protocol, which has been approved by the Ethics Review Committee of Sanbo Brain Hospital of Capital Medical University (SBNK‐YJ‐2024‐005‐01) and registered on the clinical trial registration platform (clinicaltrials.gov, NCT06480162). A waiver of informed consent was granted for the use of de‐identified data and secondary analysis (File S1). All animal procedures were approved by the Institutional Animal Care and Use Committee of Capital Medical University (AEEI‐2024‐391). Data acquisition and processing followed pre‐specified protocols across species [20]. Human research was conducted in accordance with the principles of the Declaration of Helsinki, and all animal experiments adhered to institutional and national guidelines for the care and welfare of laboratory animals. This manuscript follows the STROBE and ARRIVE guidelines.

**FIGURE 1:**
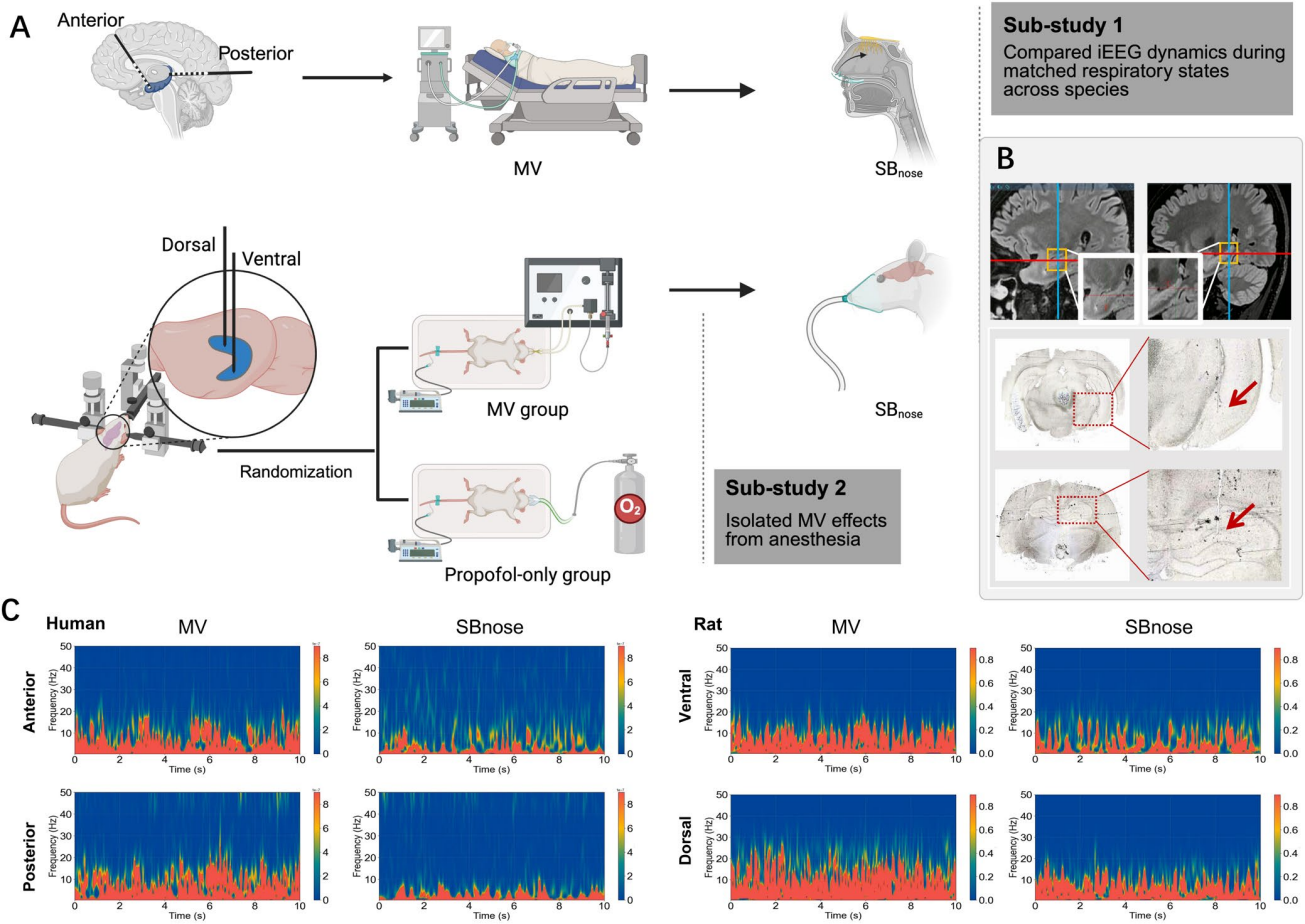
Study protocol, electrode placement, and hippocampal activity during MV and SB_nose_. (A) Schematic of the experimental protocol. (B) Electrode placement in the hippocampus: Anterior and posterior regions in humans confirmed by MRI, and ventral and dorsal regions in rats confirmed through paraffin embedding of brain sections. (C) Representative time‐frequency plots of hippocampal activity in humans (anterior and posterior) and rats (ventral and dorsal) during MV and SBnose. MV, mechanical ventilation; SBnose, spontaneous breathing with nasal airflow.

Two sub‐studies were performed [Figure 1]. The first compared iEEG dynamics after ~2 h of MV in both humans and rats, using post‐extubation spontaneous nasal breathing (SB_nose_) as a reference in each species. The second sub‐study isolated the effects of MV from anesthesia by randomizing rats to MV with propofol (MV group) or SBnose with an equivalent propofol dose (propofol‐only group).

### 2.2 Human Participants

Nine epilepsy patients (≥ 18 years) undergoing stereotactic hippocampal electrode implantation and ICU weaning from MV were included. Six patients had a unilateral anterior or posterior hippocampal electrode, while three had bilateral electrodes. Details of anesthesia and ICU care are provided in Supporting Information (File S2). The analysis used data collected during periods when patients were under volume‐controlled ventilation (tidal volume: 6 mL/kg of ideal body weight, PEEP = 5 cmH_2_O, respiratory rate = 10 bpm, setting FiO_2_ 45% for SPO_2_ > 95%). The Richmond Agitation‐Sedation Scale (RASS) was maintained within the range of −4 to −5. Continuous iEEG was recorded using a clinical acquisition system (RX40 Plus, Beike, China; sampling rate: 2048 Hz). Signals were obtained from the depth electrode contact located in the hippocampus. The left mastoid was used as the reference electrode, with the anterior forehead electrode serving as the ground.

### 2.3 Animal Model

Twelve Sprague–Dawley rats (male, 6 weeks old, 200–250 g; Charles River, China) were housed under standard conditions (24°C ± 2°C; 50% ± 10% humidity; 12‐h light/dark cycle) with ad libitum food and water. Under isoflurane anesthesia (3%–4% induction, 1.5% maintenance), tungsten electrodes (diameter 300 μm, A‐M Systems) were implanted into dorsal (analogous to human posterior; AP −3.3 mm, ML +1.8 mm, DV −3.0 mm) and ventral (human anterior; AP −4.8 mm, ML +5.2 mm, DV −8.0 mm) hippocampus [21]. After 7 ~ 10 days of recovery, rats underwent intravenous propofol anesthesia (20 mg/kg induction; 4 mg/kg/h maintenance) with the sedation level comparable to a RASS score of −4 to −5 in humans (i.e., the absence of visual response and lack of reaction to toe‐pinch stimuli). MV + propofol rats were intubated and ventilated (tidal volume 4–6 mL/kg; PEEP 0 cmH_2_O; RR 70 breaths/min). Propofol‐only rats received face mask oxygen with a matched propofol dose. Body temperature (~37°C) and heart rate and respiratory rate were continuously monitored; arterial blood gases were sampled every 2 h through the tail artery (EPOC reader Epocal Inc., Canada). Hippocampal iEEG was recorded throughout (BIOPAC MP160; 2000 Hz sampling; skull screw reference) in a noise‐controlled environment. Extubation was performed after return of spontaneous breathing, following routine criteria [22].

### 2.4 Data Analysis

Raw iEEG data were preprocessed using the MNE‐Python toolbox (v3.13) with a 0.1–50 Hz bandpass filter. Signals were decomposed into standard frequency bands: Delta (0.5–4 Hz), theta (4–7 Hz), alpha (8–12 Hz), beta (13–30 Hz), and gamma (30–47 Hz) [23, 24, 25]. Two respiratory conditions were analyzed: MV and SB_nose_. For each stage, two independent 90‐s segments were extracted according to a predefined pipeline (File S3). The selected human segments corresponded to the period immediately after ICU admission during controlled MV, prior to any ventilator parameter adjustments. The corresponding segments in rats were taken at the end of the 2‐h mechanical ventilation period, matched for depth of sedation and duration of MV. In rats, propofol‐only and MV groups were compared to isolate MV‐specific effects.

#### 2.4.1 Power Spectral Density and Frequency Band Analysis

Power spectral density (PSD) was estimated using Welch's method with 50% overlapping segments (length = 5 × sampling rate) to improve spectral stability and frequency resolution. Time‐frequency analyses were performed using Morlet wavelet transforms. Scale‐free neural dynamics were quantified by calculating the power‐law spectral exponent (SE, β), defined as the slope of the aperiodic component of the power spectrum, obtained from log–log linear fits of PSD over the 0.5–50 Hz frequency range with 1/f weighting to reduce heteroscedasticity ($\log_{10}\left(\mathrm{PSD}\right)=-\beta \cdot \log_{10}\left(f\right)$). SE reflects broadband neural activity arising from the aperiodic component of the signal.

#### 2.4.2 Nonlinear Signal Analysis

To capture neural complexity beyond traditional spectral metrics, trajectory entropy (TE) was computed. Signals (0.5–50 Hz) were resampled to 250 Hz and reconstructed in phase‐space using time‐delay embedding (embedding dimension = 4; delay = 20 ms). Entropy was calculated from Euclidean distances between successive state vectors within 200‐ms sliding windows, using permutation patterns (dimension = 4), and Shannon entropy of ordinal distributions (log₂‐normalized). To assess the parameter robustness, sensitivity analyses were conducted by varying embedding dimension (4 and 5), time delay (20 and 40 ms), and sliding window size (200 and 320 ms) within commonly used parameter ranges (Table S1). EEG amplitudes were z‐score normalized (baseline‐derived mean (μ) and standard deviation (σ)), enabling consistent group‐level comparisons ($z=\left(x-\mu \right)/\sigma$).

Clinically, TE reflects moment‐to‐moment fluctuations in neural activity, revealing subtle changes in cortical adaptability or arousal [26]. Reduced entropy indicates altered neural dynamics and may offer a potential electrophysiological marker of MV‐associated neural state modulation.

### 2.5 Statistical Analysis

Normality of iEEG metrics was tested using the Shapiro–Wilk test. Data are reported as mean ± standard deviation (SD), median [interquartile range, IQR], or number (%), as appropriate. Within‐species group comparisons (MV vs. SB_nose_) in both humans and rats were performed using paired Wilcoxon signed‐rank tests. Cross‐species analyses were performed using linear mixed‐effects models (LMMs) with subject as a random effect, and fixed effects for species, respiratory condition, anatomical location, and their interaction. Cohen's d effect sizes (R vs. H) were calculated stratified by anatomical location to quantify cross‐species differences. Complementary Bayesian mixed‐effects analyses were conducted to compute Bayes factors (BF_01_), assess the robustness of observed effects, and mitigate potential inflation of effect sizes in small‐sample cross‐species comparisons (null hypothesis: No difference between conditions). No formal sample size calculation was conducted. However, sample sizes were based on previous similar studies that compared EEG characteristics across species [27]. Group differences were evaluated using Kruskal–Wallis or Mann–Whitney U tests. Statistical significance was defined as *p* < 0.05. All analyses and visualizations were performed in Python (v3.13) and GraphPad Prism (v10.1.2).

## 3 Results

Hippocampal iEEG was recorded from nine patients (33 ± 6.3 years; 5 male patients) and twelve rats [*n* = 6 per group; Figure 1]. Human recordings included six anterior and six posterior sites, with three patients contributing both. Human demographics and respiratory parameters are summarized in (Tables 1 and S2). In brief, the differences in respiratory parameters between MV and SB_nose_ were comparable in humans (SpO_2_ MV 99.1 ± 0.6 vs. SB_nose_ 99.3 ± 0.5, *p* = 1.000; EtCO_2_ 38.5 ± 1.8 vs. 39 ± 1.1 mmHg, *p* = 0.219) and rats (PaO_2_ 97.2 ± 1.1 vs. 94.9 ± 2.2 mmHg, *p* = 0.152; PaCO_2_ 42.6 ± 1.4 vs. 46.6 ± 1.8 mmHg, *p* = 0.115). The coefficient of variation (CV) results of all electrophysiological signals are shown in Table S3.

**TABLE 1:** Respiratory parameters across these phases.

Parameters	Human		*p*‐value
	MV (*n* = 6)	SB_nose_ (*n* = 6)	
MV duration, min	125.6 ± 38.4	—	—
Respiratory rate, bpm	10	17.8 ± 4.1	< 0.001
Tidal volume, ml/kg	7	—	—
I:E ratio	1:2	—	—
SPO_2_, %	99.1 ± 0.6	99.3 ± 0.5	1.000
EtCO_2_, mmHg	38.5 ± 1.8	39 ± 1.1	0.219
	**Rat**		
	**MV (*n* = 6)**	**SB_nose_ (*n* = 6)**	
MV duration, min	120	120	1.000
Respiratory rate, bpm	70	63.2 ± 2.93	< 0.001
Tidal volume, ml/kg	4–6	—	—
I:E ratio	1:1	—	—
PaO_2_, mmHg	97.2 ± 1.1	94.9 ± 2.2	0.152
PaCO_2_, mmHg	42.6 ± 1.4	46.6 ± 1.8	0.115
	**Rat**		
	**MV group (*n* = 6)**	**Propofol‐only group (*n* = 6)**	
MV duration, min	120	—	1.000
Respiratory rate, bpm	70	66.4 ± 3.93 ^##^	< 0.001
Tidal volume, ml/kg	4–6	—	—
I:E ratio	1:1	—	—
PaO_2_, mmHg	97.2 ± 1.1	96.6 ± 2.1	0.514
PaCO_2_, mmHg	42.6 ± 1.4	45.7 ± 2.2	0.065

Abbreviations: MV, mechanical ventilation; SB_nose_, spontaneous nasal breathing. Within‐species group comparisons (MV vs. SB_nose_) in both humans and rats used paired Wilcoxon signed‐rank tests. Group differences were evaluated using Kruskal–Wallis or Mann–Whitney U tests.

### 3.1 Humans Hippocampal iEEG During MV and SB_nose_ Conditions

**FIGURE 2:**
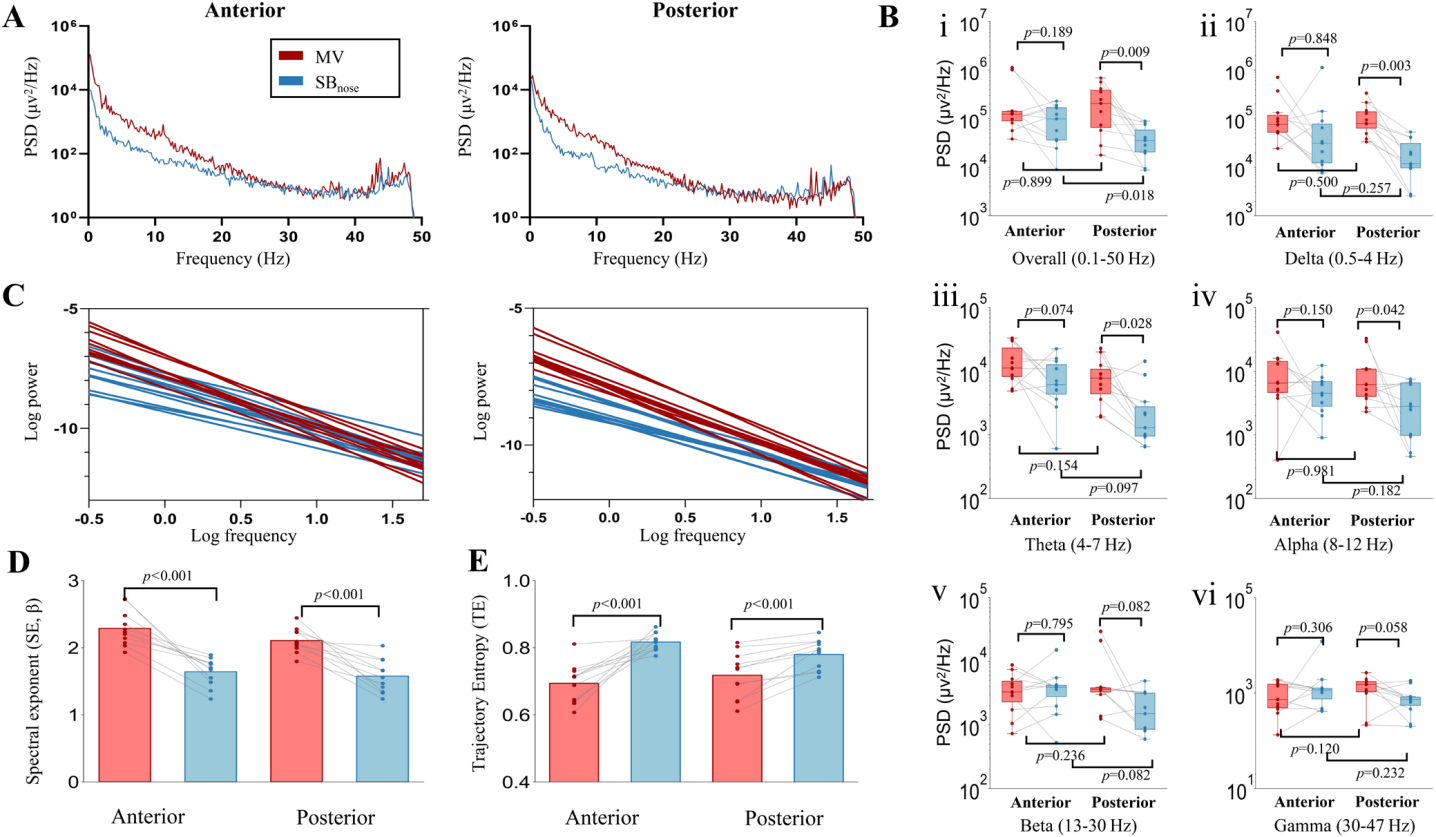
Human hippocampal iEEG during MV and SB_nose_. (A) Group‐averaged power spectral density (PSD) of hippocampal iEEG under MV (red) and SB_nose_ (blue) in anterior (left) and posterior (right) hippocampus. (B) Box plots compare PSD across respiratory conditions and regions, including overall frequency band (i) and delta‐gamma bands (ii–vi). (C) Representative log–log plots of the power‐law spectral exponent (SE, β) from anterior and posterior hippocampus. (D) Column plots compare SE between MV and SB_nose_ across anterior and posterior sites. (E) Column plots of trajectory entropy across conditions in anterior and posterior sites. Statistical analysis used ANOVA or non‐parametric alternatives (Kruskal–Wallis or Mann–Whitney U tests). Each data point represents one 90‐s segment, with two independent segments analyzed per patient. Paired data points across conditions are connected by gray lines.

MV increased overall PSD (×10^5^ μV^2^/Hz), reaching statistical significance in the posterior hippocampus (MV 1.83 [0.62–3.99] vs. SB_nose_ 0.36 [0.19–0.67], *p* = 0.009) [Figure 2A,B (i)]. Power increases were concentrated in the low‐frequency range and primarily localized to the posterior hippocampus [Figure 2 B(ii–vi)]. The posterior hippocampus showed significant increases in delta (MV 0.78 [0.57–1.43] vs. SB_nose_ 0.14 [0.10–0.36], *p* = 0.003), theta (MV 0.08 [0.04–0.12] vs. SB_nose_ 0.02 [0.01–0.03], *p* = 0.028), and alpha (MV 0.06 [0.04–0.11] vs. SB_nose_ 0.03 [0.01–0.07], *p* = 0.042).

SE (a.u.) increased during MV in both anterior (MV 2.22 [2.04–2.45] vs. SB_nose_ 1.69 [1.39–1.77], *p* < 0.001) and posterior hippocampus (MV 2.08 [2.01–2.27] vs. SB_nose_ 1.51 [1.36–1.78], *p* < 0.001) [Figure 2C,D]. TE was reduced during MV (anterior: 0.71 [0.64–0.73] vs. 0.81 [0.79–0.84], *p* < 0.001; posterior: 0.72 [0.65–0.77] vs. 0.79 [0.73–0.82], *p* < 0.001) [Figure 2E], consistent with altered neural dynamics. There was a significant difference between the anterior and posterior hippocampal regions in PSD (*p* = 0.018). However, no significant differences were observed between the anterior and posterior hippocampus across other metrics. More details are presented in Table S4.

### 3.2 Rat Hippocampal iEEG During MV and Spontaneous Breathing

**FIGURE 3:**
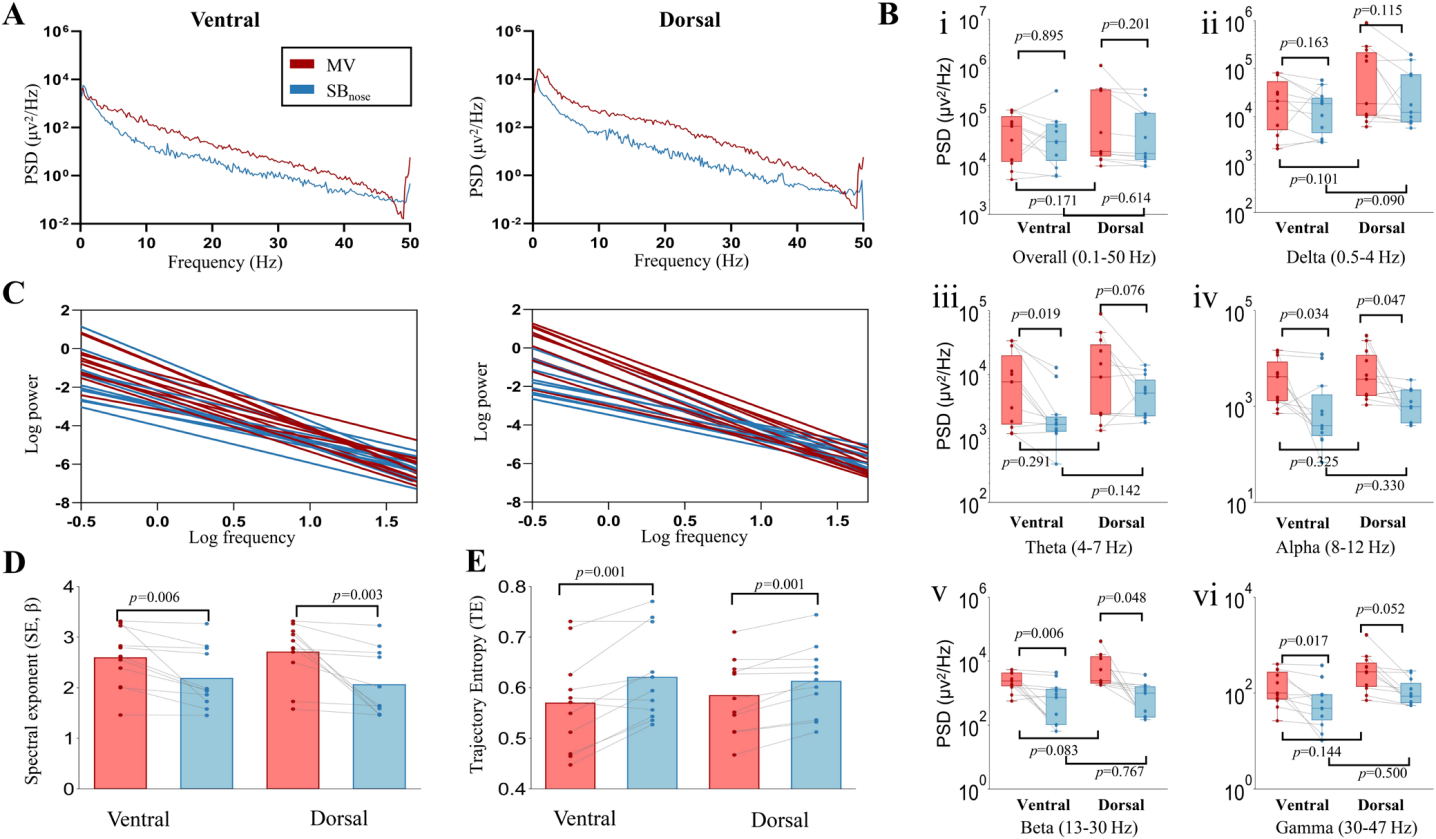
Rat hippocampal iEEG during MV and SB_nose_. (A) Group‐averaged PSD under MV (red) and SB_nose_ (blue) in ventral (left) and dorsal (right) hippocampus. (B) Box plots compare PSD across conditions and regions, including overall frequency band (i) and delta‐gamma bands (ii–vi). (C) Representative log–log plots of power‐law spectral exponent (SE, β) from ventral and dorsal hippocampus. (D) Column plots compare SE between MV and SB_nose_ across ventral and dorsal sites. (E) Column plots of trajectory entropy across conditions in ventral and dorsal sites. Statistical analysis used ANOVA or non‐parametric alternatives (Kruskal–Wallis or Mann–Whitney U tests). Each data point represents one 90‐s segment, with two independent segments analyzed per patient. Paired data points across conditions are connected by gray lines.

MV increased overall PSD, though not statistically significant [Figure 3A,B (i)]. Sub‐band analyses revealed significant PSD (×10^3^ μV^2^/Hz) elevation in the ventral hippocampus during MV, in theta (MV 5.35 [1.57–23.95] vs. SB_nose_ 1.53 [1.21–2.26], *p* = 0.019), alpha (MV 2.86 [0.97–10.11] vs. SB_nose_ 0.37 [0.20–2.21], *p* = 0.034), beta (MV 2.15 [1.04–4.28] vs. SB_nose_ 0.48 [0.10–1.38], *p* = 0.006) and gamma (MV 0.10 [0.06–0.29] vs. SB_nose_ 0.05 [0.05–0.09], *p* = 0.017). The dorsal hippocampus showed significant alpha and beta band increases (Table S4).

SE was significantly elevated during MV in both ventral (MV 2.59 [2.11–3.12] vs. SB_nose_ 1.95 [1.66–2.75], *p =* 0.006) and dorsal hippocampus (MV 2.78 [2.52–3.10]) vs. SB_nose_ 1.66 [1.50–2.67], *p* = 0.003 [Figure 3C,D]. TE showed a similar significant decrease during MV. [Figure 3E]. No significant differences were observed between ventral and dorsal hippocampus across regional metrics (Table S4).

#### 3.2.1 Cross‐Species Comparison Under MV

**FIGURE 4:**
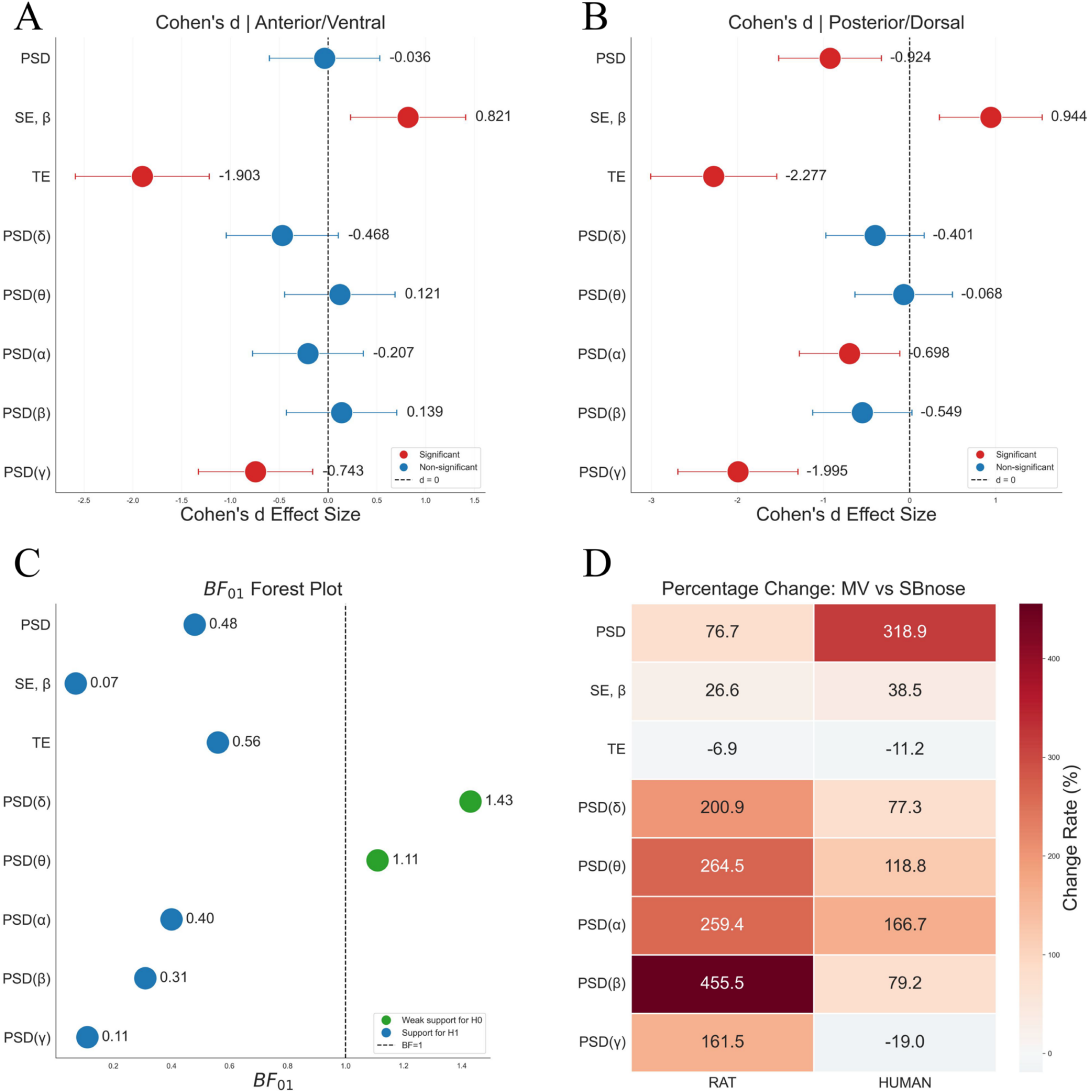
Effect size comparison of MV and SB_nose_ across species and hippocampal regions. (A) Forest plots show the effect sizes (Cohen's d) for power spectral density (PSD), spectral exponent (SE), trajectory entropy (TE), and sub‐band PSD across anterior/ventral hippocampal regions of rats and humans. (B) Forest plots show effect sizes (Cohen's d) for PSD, SE, TE, and sub‐band PSD across posterior/dorsal hippocampal regions of rats and humans. (C) Forest plots present Bayes Factors (BF_01_) for PSD, SE, TE, and sub‐band PSD. (D) Heatmaps display the percentage change in PSD, SE, TE, and sub‐band PSD under MV compared with SB_nose_, across rats and humans. Color intensity represents the percentage of change, with gray indicating reductions and red indicating increases relative to the SB_nose_.

Across both humans and rats, MV was associated with increased PSD and SE and decreased TE. PSD increased in humans (318.9%) and rats (76.7%), SE increased in humans (38.5%) and rats (26.6%), while TE decreased in humans (11.2%) and rats (6.9%). Sub‐band analysis showed broadly consistent low‐frequency modulation, although the distribution of affected frequency bands varied between species [Figure 4D].

Species‐specific differences were mainly reflected in effect magnitude and regional specificity. PSD changes were more pronounced in the posterior/dorsal hippocampus (Cohen's d = −0.924 [−1.521 to −0.328]) than in anterior/ventral regions (d = −0.036 [−0.601 to 0.530]), supported by Bayesian evidence (BF₀₁ = 0.48). Similarly, SE and TE showed stronger divergence in both anterior/ventral (SE: d = 0.821; TE: d = −1.903) and posterior/dorsal regions (SE: d = 0.944; TE: d = −2.277) [Figure 4A–C]. Sub‐band analyses confirmed no robust species differences in δ and θ activity (BF₀₁ > 1), whereas α and γ bands showed divergence, particularly in posterior/dorsal hippocampus (α: d = −0.698; γ: d = −1.995). Rats showed more uniform broadband increases, while humans exhibited more selective high‐frequency modulation.

#### 3.2.2 Effects of MV Independent of Propofol

**FIGURE 5:**
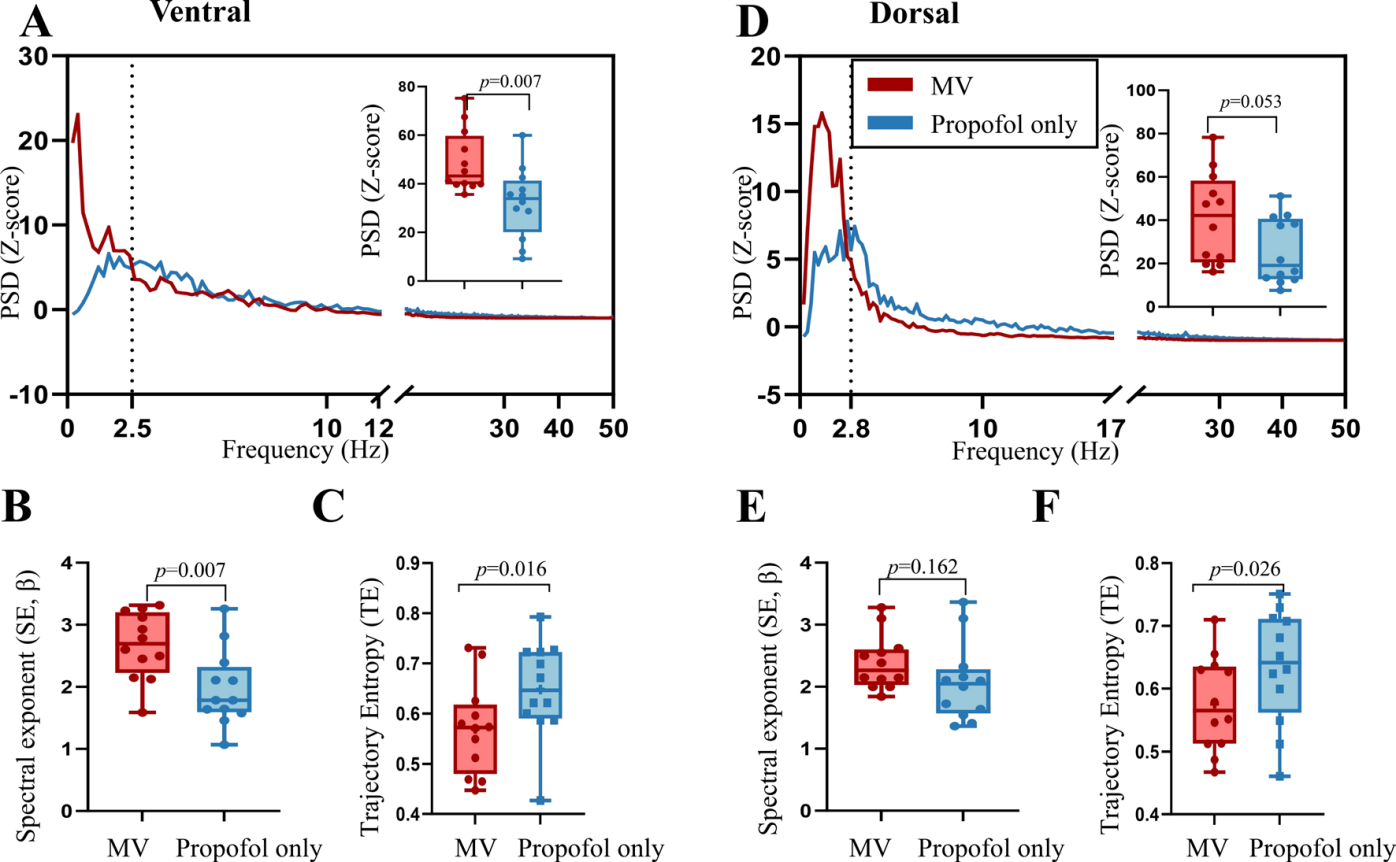
Effects of MV independent of propofol. (A) Group‐averaged PSD under MV (red) and Propofol‐only (blue) in ventral hippocampus; inset box‐plots show differences between the two conditions. (B) Column plots compare SE (B) between MV and Propofol‐only in the ventral hippocampus. (C) Column plots compare TE between MV and Propofol only in the ventral hippocampus. (D) Group‐averaged PSD under MV (red) and Propofol‐only (blue) in dorsal hippocampus; inset box‐plots show the differences between the two conditions. (E) Column plots compare SE between MV and Propofol only in the dorsal hippocampus. (F) Column plots compare TE between MV and Propofol only in the dorsal hippocampus.

Compared with the propofol‐only condition, MV significantly altered hippocampal power but showed region‐dependent effects on complexity measures. Specifically, MV increased low‐frequency power, with crossover points at 2.5 Hz (ventral) and 2.8 Hz (dorsal) (ventral: MV 43.22 [39.75–59.69] vs. Propofol‐only 33.89 [20.11–41.32], *p* = 0.007; dorsal: MV 42.18 [20.53–58.27] vs. Propofol‐only 19.11 [12.83–40.72], *p* = 0.053; Figure 5, Table S5).

For SE, MV significantly increased values in the ventral hippocampus (MV 2.69 [2.22–3.20] vs. propofol‐only 1.78 [1.59–2.32], *p* = 0.007) but not in the dorsal (MV 2.27 [2.03–2.60] vs. propofol‐only 2.05 [1.57–2.28], *p* = 0.162) hippocampus. Moreover, TE showed a significant decrease in both ventral (MV 0.57 [0.48–0.62] vs. propofol‐only 0.65 [0.59–0.72], *p* = 0.016) and dorsal (MV 0.57 [0.51–0.64] vs. propofol‐only 0.64 [0.56–0.71], *p* = 0.026) hippocampus.

## 4 Discussion

In the present study, we investigated hippocampal electrophysiological responses to short‐term MV using iEEG recordings in both humans and rats. Our results show that short‐term MV is associated with increased hippocampal spectral power and SE, alongside reduced TE, indicating altered local activity and network‐level information flow. Notably, these effects were observed across species and under controlled propofol anesthesia conditions, suggesting consistent neural responses associated with MV. Given that our study focused on electrophysiological measurements without assessing molecular, structural, or behavioral injury markers, these findings should be interpreted as evidence of functional neural modulation or increased neural susceptibility rather than direct proof of brain injury. Nevertheless, the consistent hippocampal dynamics observed during MV across species highlight early electrophysiological signatures of MV‐associated modulation and suggest the potential translational value of rodent models for investigating the neural mechanisms underlying MV‐associated neural effects.

Across species, MV was associated with increased power in hippocampal oscillations and an elevation of SE, reflecting alterations in the aperiodic component of the EEG and overall network dynamics. While SE has been linked to excitation‐inhibition balance in previous studies [28, 29, 30], this interpretation remains debated. Therefore, we interpret the observed changes more cautiously as reflecting broader changes in population‐level neural activity rather than direct evidence of increased inhibition. Further studies are required to clarify the underlying neurophysiological mechanisms driving these effects. Complementary reductions in TE suggested reduced directed information flow and changed dynamic variability during MV [29]. Although entropy‐based measures may be influenced by global brain state, including anesthesia depth and physiological variability, the consistency of these findings across species and experimental conditions suggests a stable pattern of MV‐associated modulation rather than nonspecific fluctuation. These electrophysiological signatures align with preclinical reports linking MV to neurophysiological alterations and extend these findings by demonstrating similar patterns in the human hippocampus during clinically relevant ventilation conditions [10, 11, 12, 13]. Importantly, the observed changes occurred in the absence of major alterations in systemic oxygenation or CO_2_ levels, indicating that they likely arise from MV‐associated physiological coupling rather than respiratory derangement.

Cross‐species, MV was associated with largely similar directional changes in PSD, SE, and TE, although the magnitude and frequency distribution varied between species. This is supported by the statistical results, which show robust effects for SE and TE across all regions, whereas PSD effects were more region‐ and species‐dependent, reaching significance primarily in the posterior/dorsal hippocampus and in specific frequency bands (e.g., posterior human and ventral rat hippocampus). Bayesian analysis showed consistent across‐species results for all these parameters, especially in the anterior/ventral region, indicating the consistency of the findings.

Consistent with this pattern, SE showed stronger and more widespread effects than PSD across both species, particularly in posterior/dorsal regions where multiple frequency bands also reached significance. In contrast, anterior/ventral hippocampal PSD changes were largely non‐significant, indicating spatial heterogeneity of spectral power modulation, warranting validation in larger sample cohorts. Humans exhibited more prominent low‐frequency augmentation, whereas rats demonstrated broader band involvement, including effects extending into higher‐frequency bands, particularly in the ventral hippocampus. Notably, these higher‐frequency changes in rats should not be interpreted as evidence of increased neural complexity; rather, they likely reflect altered local synchronization or changes in network state under MV. These regional and species differences are consistent with known anatomical and functional distinctions between rodent and human hippocampus [31, 33].

Nevertheless, the consistent directional changes in entropy‐based measures across species indicate a shared pattern of MV‐associated modulation. However, frequency‐domain effects were more heterogeneous across regions and species, indicating that band‐specific responses may be influenced by both biological and methodological factors.

In addition, the randomized rat experiment allowed us to isolate the effects of MV from propofol anesthesia. Propofol depth was titrated to a sedation depth comparable to the human RASS range observed during MV, with behavioral suppression assessed using reflex‐based toe‐pinch response. Under these matched conditions, MV selectively increased low‐frequency power and reduced high‐frequency activity, with region‐specific crossover patterns observed in the dorsal and ventral hippocampus.

In rats, TE showed consistent modulation across regions, whereas SE effects were region‐dependent, with a significant reduction in the ventral hippocampus but not in the dorsal hippocampus. These results indicate that TE represents the most stable cross‐condition marker of MV‐related modulation under controlled anesthesia, while SE exhibits spatially heterogeneous sensitivity.

Although SE and TE showed directional consistency with the human findings, their statistical strength varied across hippocampal subregions, supporting cautious interpretation of cross‐species convergence. Moreover, baseline TE values differed between humans and rats, reflecting inherent interspecies neurophysiological and structural heterogeneity. Therefore, absolute TE values are not directly comparable across species, and interpretation should focus on relative within‐species changes.

These results suggest that MV under controlled sedation conditions is associated with alterations in hippocampal oscillatory organization. However, further studies are required to confirm these findings and explore the underlying mechanisms by which MV impacts sedated subjects. SE and TE should be interpreted as early, process‐based electrophysiological markers reflecting neural state dynamics rather than direct predictors of clinical outcomes. Their value lies in capturing neurodynamic changes before brain dysfunction becomes clinically apparent. As reported in previous studies [33, 34], these metrics are associated with cortical excitation‐inhibition balance and network organization. In this context, changes in SE and TE may serve as real‐time indicators of acute neurophysiological modulation during MV. However, they should not be considered definitive predictors of long‐term cognitive impairment without longitudinal behavioral validation.

Collectively, our findings demonstrate that short‐term MV is associated with reproducible alterations in hippocampal oscillatory activity and SE and TE across species under controlled anesthetic and physiological conditions. While these findings suggest measurable modulation of hippocampal networks, directional consistency rather than absolute equivalence should be emphasized, given anatomical and functional differences between human and rat hippocampi. Replication across species, multiple independent metrics (PSD, SE, TE), and harmonized signal processing support robustness, though methodological and scaling factors cannot be fully excluded. Thus, causal attribution to specific lung–brain pathways remains inferential.

Multiple pathways of lung‐brain communication have been described in prior literature, including vagal afferent signaling from pulmonary mechanoreceptors, modulation of respiratory‐neural coupling, autonomic regulation via cholinergic and catecholaminergic systems, inflammatory mediator release, and chemoreceptor‐driven influences on central networks [3, 12, 35, 36, 37, 38, 39, 40, 41, 42]. These mechanisms offer biologically plausible frameworks that are compatible with our observations; nevertheless, they were not directly interrogated in the present study and therefore remain hypothetical within this context. Future investigations employing pathway‐specific interventions, targeted physiological monitoring, and mechanistic perturbation paradigms will be necessary to delineate the relative contribution of these processes to ventilation‐associated neural modulation.

Several limitations must be acknowledged. First, the human cohort consisted only of epilepsy patients. Although recordings were obtained from non‐epileptogenic hippocampal contacts during stable monitoring periods, disease‐related network alterations, antiseizure medication, and postoperative factors may have influenced hippocampal activity. At the same time, intracranial hippocampal recordings during MV are rarely available in humans, providing a unique opportunity to examine MV‐associated neural dynamics directly. Second, the sample size was modest, particularly for cross‐species comparisons. While this is an inherent challenge in studies involving invasive human hippocampal recordings, the main findings remained consistent across multiple recording epochs and were observed in both humans and rats. Nevertheless, larger studies will be important for refining effect‐size estimates and characterizing inter‐individual variability. Third, differences in propofol exposure between clinical conditions may have contributed to the observed electrophysiological changes. Although this confounding factor cannot be fully excluded in the human cohort, the complementary rat experiments were designed to evaluate MV‐associated effects under more controlled anesthetic conditions. Finally, the study examined only short‐term (~2 h) MV and did not include molecular, inflammatory, histological, behavioral, or cognitive assessments. Therefore, the observed changes should be interpreted as MV‐associated electrophysiological modulation rather than evidence of neural injury or long‐term dysfunction. Nonetheless, the cross‐species framework developed here may provide a useful foundation for future studies aimed at linking electrophysiological alterations with underlying biological mechanisms and functional outcomes.

## 5 Conclusion

MV is associated with rapid and largely consistent changes in hippocampal oscillatory activity in both humans and rats under propofol anesthesia. These cross‐species neural alterations in EEG‐derived metrics reflect modulation of neural dynamics rather than direct evidence of brain injury. These findings support the use of a rat model for investigating MV‐associated electrophysiological brain dynamics. In addition, PSD, SE, and TE provide objective measures of neural state modulation during MV and may facilitate future mechanistic studies integrating electrophysiological, molecular, and behavioral approaches.

## Author Contributions

Study design: X.Q, B.W, Z.S. Conception: J.Z, B.W, Z.S. Accessed and verified the data: X.Q, J.L, F.X. Data acquisition: X.Q, W.S, M.Z, H.W. Data analysis: X.Q, X.O, J.W. Write the first draft of the manuscript: X.Q. Critical revision of the manuscript: Y.S, B.W, Z.S. Approval of the final version of the manuscript: All authors.

## Funding

This work was supported by the Excellent Young Talents Project of Capital Medical University (Grant No. A2308).

## Disclosure

The authors have nothing to report.

## Ethics Statement

This work has been approved by the Ethics Review Committee of Sanbo Brain Hospital of Capital Medical University (SBNK‐YJ‐2024‐005‐01).

## Conflicts of Interest

The authors declare no conflicts of interest.

## Supporting information


**File S1:** The eligibility criteria of human participants.
**File S2:** Surgery routine and ICU care of human participants.
**File S3:** iEEG segment selection method.
**Table S1:** Summary of data on TE sensitivity analysis.
**Table S2:** Demographic Characteristics of Patients Included in the Study.
**Table S3:** CV of electrophysiological signals.
**Table S4:** Data summary of different species.
**Table S5:** Data summary of different conditions.

## Data Availability

The data that support the findings of this study are available from the corresponding author upon reasonable request.
