# Supplementary material for: Mechanical Ventilation‐Associated Changes in Hippocampal Electroencephalogram: A Cross‐Species Study in Humans and Rats
Source: CNS Neurosci Ther. 2026 Jul 15;32(7):e71012. doi: 10.1002/cns.71012 (PMC13371090; doi:10.1002/cns.71012)
Supplement: Supplementary file 1 — File S1: The eligibility criteria of human participants. File S2: Surgery routine and ICU care of human participants. File S3: iEEG segment selection method. Table S1: Summary of data on TE sensitivity analysis. Table S2: Demographic Characteristics of Patients Included in the Study. Table S3: CV of electrophysiological signals. Table S4: Data summary of different species. Table S5: Data summary of different conditions. [file CNS-32-e71012-s001.docx]

**Mechanical Ventilation-Associated Changes in Hippocampal Electroencephalogram: A Cross-Species Study in Humans and Rats**

Xiang Qi MM^1,2^, Xiaoyu Ou BM^3^, Jingyi Li MM^2,4^, Meizhizi Zhang BM^2,4^, Fenqin Xue MD^5^, Hua Wei MD^5^, Wenting Su MD^2,6^, Jing Wang MD^3^, Yongxing Sun MD,PhD^1,2^, Jian Zhou MD^7,8^, Baoguo Wang MD, PhD^1,2*^, Zhonghua Shi MD, PhD^2,4,9^

^1^Department of Neurosurgery, Department of Anesthesiology, Sanbo Brain Hospital, Capital Medical University, Beijing, China

^2^Laboratory for Clinical Medicine, Capital Medical University, Beijing, China

^3^Beijing Key Laboratory of Traffic Data Mining and Embodied Intelligence, School of Computer Science and Technology, Beijing Jiaotong University, Beijing, China

^4^Department of Neurosurgery, Department of Intensive Care Medicine, Sanbo Brain Hospital, Capital Medical University, Beijing, China

^5^Laboratory of in vivo electrophysiology, Core Facility Center of Capital Medical University, Beijing, China

^6^Beijing Institute of Brain Disorders, Laboratory of Brain Disorders, Ministry of Science and Technology, Collaborative Innovation Center for Brain Disorders, Capital Medical University, Beijing, China

^7^Epilepsy Center, Sanbo Brain Hospital, Capital Medical University, Beijing, China

^8^Department of Neurosurgery, Sanbo Brain Hospital, Capital Medical University, Beijing, China

^9^ Department of Physiology, Amsterdam University Medical Centre, Amsterdam, The Netherlands

**Corresponding author:**

Baoguo Wang, PhD, MD,

Department of Neurosurgery, Department of Anesthesiology, Sanbo Brain Hospital, Capital Medical University, Beijing, China

E-mail address: [wangbg@ccmu.edu.cn](mailto:wangbg@ccmu.edu.cn)

## File S1. The eligibility criteria of human participants

**Inclusion Criteria:**

- Age: ≥18 years
- ASA: I-II
- W/electrode inside hippoampus (≥1 contacts)
- Singed consent form

**Exclusion Criteria:**

- Seizure occurrence≤36 hours before operation.
- Structural brain damage confirmed by CT/MRI.
- History of using: opioids, enzyme-inducing medications,sleep aids, or excessive alcohol consumption
- History of mechanical ventilation(>24h) within 6 months
- Cognitive impairment (Mini-Mental State Examination <24).
- Craniotomy within 6 months
- Participate in other clinical trials in the last four weeks
- Pregnant or breastfeeding

## File S2. Surgery routine and ICU care of human participants

Intracranial electrode placement surgery was performed under general anesthesia following a standardized protocol. In brief, anesthesia induction was achieved with an intravenous infusion of 1% propofol at a target concentration of 3.5μg/ml (target-controlled infusion, TCI-I, Guangxi VERYARK Technology Co., Ltd, China), remifentanil (0.3μg/kg), and rocuronium (7mg/kg) to facilitate muscle relaxation. Anesthesia was maintained with propofol (3.5μg/ml) and remifentanil (0.2μg/kg/min). No additional neuromuscular blockers were required due to the short duration of procedure.

After patient positioning in a stereotactic frame, the surgical site was sterilized, and electrode placement was guided by preoperative imaging and neuronavigation for precise localization. Electrodes with 16 cylindrical platinum-iridium contacts (0.8 mm diameter, Huake Hengsheng Medical Technology Co. Ltd., Beijing, China) were implanted, with intraoperative iEEG monitoring confirming placement. Following successful electrode implantation and verification, the head was wrapped in a sterile dressing, leaving the electrode wires exposed for continued monitoring. Postoperatively, patients were transferred to the intensive care unit (ICU) for ventilator weaning, continuous iEEG monitoring, and stabilization. Electrode contact positions were further confirmed through postoperative fusion of post-operative CT and MRI scans to ensure proper electrode placement.

In ICU, continuous monitoring was performed for heart rate, respiratory rate, SpO_2_, end-tidal CO_2_ (EtCO_2_), and temperature to assess the patient's physiological status. Additionally, non-invasive blood pressure was measured, and a comprehensive neurological physical examination was conducted at 5-minute intervals. This examination included the assessment of consciousness level, pupil size and light reflex, and the Richmond Agitation-Sedation Scale (RASS) to monitor sedation and neurological function.

Extubation was performed based on the clinical judgment of the attending physician, considering factors such as the patient’s respiratory stability, neurological status, and overall readiness for extubation.

## File S3. iEEG segment selection method

The following outlines the segment selection pipeline used for extracting 90 second segments from continuous iEEG recordings, with total recording durations ranging between 60 to 100 minutes. This pipeline is designed for the extraction of high-quality data segments suitable for subsequent analysis.

1. Data Preparation

The iEEG data, recorded using standard clinical or experimental protocols, were exported in a common format (edf) and loaded into the analysis environment using appropriate data-processing libraries.

2. Preprocessing

To ensure high data quality, the following preprocessing steps were performed:

Artifact Removal: Obvious artifacts (e.g., muscle or electrical interference) were removed manually.

Filtering: The data was bandpass filtered between 0.1 Hz and 50 Hz to remove low-frequency drift and high-frequency noise, which could interfere with the analysis.

3. Segment Selection

For each recording, two independent 90 second segments were selected for analysis. The selection process involved the following steps:

Selection of Start Time: A starting time was chosen as the 5 minutes prior to propofol discontinuation for the MV condition. and for the SBnose condition, segments were selected starting at the time of extubation.

Segment Extraction: Two independent 90 second segments were selected consecutively in time.

4. Data Extraction

Using the software environment (Python), the desired segment was extracted by cropping the continuous iEEG signal. The data segment was saved as a new file for subsequent analysis.

5. Quality Control

Following segment extraction, each 1-minute segment was visually inspected for artifacts or abnormalities. A combination of time-domain inspection and frequency-domain analysis (e.g., power spectral density) was used to assess the quality and reliability of each segment. If any irregularities were detected (e.g., large spikes, muscle artifacts), the segment was discarded, and a new segment was selected.

6. Saving and Exporting

Once validated, the extracted 1-minute iEEG segment was saved in the desired format (edf) for use in further analysis.

| Table S1. Summary of data on TE sensitivity analysis | | | |
| --- | --- | --- | --- |
|  | **Human** | | **P-value** |
|  | MV | SB_nose_ |  |
| Anterior | 0.71(0.70-0.73) | 0.78(0.76-0.80) | <0.001 |
| Posterior | 0.70(0.69-0.76) | 0.79(0.74-0.81) | 0.015 |
|  | **Rat** | | **P-value** |
|  | MV | SB_nose_ |  |
| Ventral | 0.56（0.51-0.61） | 0.62（0.55-0.66） | 0.165 |
| Dorsal | 0.56（0.51-0.65） | 0.60（0.54-0.66） | 0.489 |

Data (embedding dimension=5, delay=40ms, or window size=320ms) are presented as median, (quartile Q1-Q3). Statistical analysis was performed using paired Wilcoxon signed-rank tests. Abbreviations: MV, mechanical ventilation; SB_nose_, spontaneous nasal breathing

##

## Table S2. Demographic Characteristics of Patients Included in the Study

| Variables | Patient 1 | Patient 2 | Patient 3 | Patient 4 | Patient 5 | Patient 6 | Patient 7 | Patient 8 | Patient 9 |
| --- | --- | --- | --- | --- | --- | --- | --- | --- | --- |
| Gender, F/M | F | F | M | M | F | M | M | F | M |
| Age, year | 37 | 32 | 23 | 32 | 28 | 42 | 31 | 30 | 42 |
| Body Mass Index, kg/m^2^ | 24.2 | 27.2 | 17.5 | 22.7 | 24.2 | 24.2 | 29.1 | 23.6 | 25.1 |
| Onset of epilepsy, age | 25 | 12 | 15 | 17 | 9 | 19 | 24 | 25 | 12 |
| Duration of surgery, hours | 1.6 | 2.3 | 1.6 | 2.1 | 1.3 | 2.3 | 0.92 | 1.17 | 1.25 |
| Confirmed foci of epilepsy after surgery |  |  |  |  |  |  |  |  |  |
| Occipital lobe | √ |  | √ |  |  | √ | √ |  |  |
| Temporal lobe |  | √ |  | √ | √ |  |  | √ | √ |
| Propofol concentration (TCI), μg/ml |  |  |  |  |  |  |  |  |  |
| MV | 3.5 | 3.5 | 3.5 | 3.5 | 3.5 | 3.5 | 3.5 | 3.5 | 3.5 |
| SB_nose_ | 1.1 | 1.2 | 0.9 | 1.0 | 1.1 | 1.0 | 0.9 | 1.0 | 1.3 |
| Semi-hippocampus for iEEG data collection | anterior | both | anterior | posterior | both | both | posterior | anterior | posterior |

Abbreviations: F, female; M, male; TCI, target-controlled infusion; iEEG, intracranial electroencephalogra; MV, mechanical ventilation; SB_nose_, spontaneous nasal breathing.

**Table S3. CV of electrophysiological signals**

| **Location** | **Metrics** | **CV_(Human)_** | | **CV_(Rat)_** | |
| --- | --- | --- | --- | --- | --- |
|  |  | **MV** | **SB_nose_** | **MV** | **SB_nose_** |
| Anterior/Ventral | Overall PSD | 0.27 | 1.13 | 3.55 | 1.53 |
|  | δ PSD | 0.51 | 1.68 | 0.94 | 0.92 |
|  | θ PSD | 0.69 | 1.11 | 1.80 | 1.13 |
|  | α PSD | 1.02 | 0.77 | 1.74 | 1.26 |
|  | β PSD | 0.47 | 0.44 | 2.47 | 1.59 |
|  | γ PSD | 1.09 | 0.30 | 0.87 | 0.57 |
|  | SE | 0.11 | 0.14 | 0.18 | 0.38 |
|  | TE | 0.08 | 0.03 | 0.13 | 0.14 |
| Posterior/Dorsal | Overall PSD | 0.80 | 0.58 | 1.39 | 0.79 |
|  | δ PSD | 0.58 | 0.84 | 1.14 | 0.75 |
|  | θ PSD | 0.46 | 1.04 | 1.19 | 0.38 |
|  | α PSD | 0.81 | 0.85 | 0.79 | 2.10 |
|  | β PSD | 0.22 | 1.14 | 0.39 | 1.71 |
|  | γ PSD | 0.43 | 0.21 | 0.87 | 0.86 |
|  | SE | 0.08 | 0.15 | 0.13 | 0.48 |
|  | TE | 0.08 | 0.07 | 0.16 | 0.10 |

Abbreviations: MV, mechanical ventilation; SB_nose_, spontaneous nasal breathing; SE, Power-law spectral exponent (β); TE, Trajectory Entropy. The unit of CV is dimensionless.

## Table S4. Data summary of different species

| **Location** | **Metrics** | **Human** | | | **Rat** | | |
| --- | --- | --- | --- | --- | --- | --- | --- |
|  |  | **MV** | **SB_nose_** | **P-value** | **MV** | **SB_nose_** | **P-value** |
| Anterior/Ventral | Overall PSD (×10^5^μv^2^/Hz) | 1.24(0.86-1.42) | 1.11(0.36-1.69) | 0.189 | 0.49(0.08-1.12) | 0.31(0.10-0.74) | 0.895 |
|  | δ PSD (×10^5^μv^2^/Hz) | 0.82(0.52-1.25) | 0.26(0.12-0.84) | 0.848 | 0.18(0.05-0.64) | 0.15(0.04-0.25) | 0.163 |
|  | θ PSD (×10^5^μv^2^/Hz) | 0.12(0.08-0.27) | 0.06(0.03-0.14) | 0.074 | 0.05(0.02-0.24) | 0.02(0.01-0.02) | **0.019** |
|  | α PSD (×10^4^μv^2^/Hz) | 0.64(0.44-1.49) | 0.43(0.21-0.69) | 0.150 | 0.29(0.10-1.01) | 0.04(0.02-0.22) | **0.034** |
|  | β PSD (×10^3^μv^2^/Hz) | 3.27(2.01-5.05) | 3.89(1.59-4.22) | 0.795 | 2.15(1.04-4.28) | 0.48(0.10-1.38) | **0.006** |
|  | γ PSD (×10^3^μv^2^/Hz) | 0.66(0.47-1.55) | 1.16(0.61-1.52) | 0.306 | 0.10(0.06-0.29) | 0.05(0.02-0.09) | **0.017** |
|  | SE (a.u.) | 2.22(2.04-2.45) | 1.69(1.39-1.77) | **<0.001** | 2.59(2.11-3.12) | 1.95(1.66-2.75) | **0.006** |
|  | TE (a.u.) | 0.71(0.64-0.73) | 0.81(0.79-0.84) | **<0.001** | 0.57(0.48-0.62) | 0.61(0.55-0.71) | **0.001** |
| Posterior/Dorsal | Overall PSD (×10^5^μv^2^/Hz) | 1.84(0.62-3.99) | 0.37(0.20-0.67) | **0.009** | 0.19(0.15-3.64) | 0.17(0.13-1.19) | 0.201 |
|  | δ PSD (×10^5^μv^2^/Hz) | 0.80(0.57-1.43) | 0.14(0.10-0.36) | **0.003** | 0.15(0.10-2.31) | 0.11(0.07-0.76) | 0.115 |
|  | θ PSD (×10^5^μv^2^/Hz) | 0.08(0.04-0.12) | 0.02(0.01-0.03) | **0.028** | 0.06(0.02-0.32) | 0.04(0.02-0.09) | 0.076 |
|  | α PSD (×10^4^μv^2^/Hz) | 0.62(0.39-1.10) | 0.27(0.10-0.67) | **0.042** | 0.28(0.16-1.29) | 0.10(0.04-0.22) | **0.047** |
|  | β PSD (×10^3^μv^2^/Hz) | 3.64(3.15-5.63) | 1.48(0.83-3.16) | 0.082 | 2.43(2.00-1.47) | 0.63(0.16-1.64) | **0.048** |
|  | γ PSD (×10^3^μv^2^/Hz) | 1.34(0.89-1.74) | 0.74(0.51-0.83) | 0.058 | 0.27(0.10-0.45) | 0.09(0.06-0.18) | 0.052 |
|  | SE (a.u.) | 2.08(2.01-2.27) | 1.51(1.36-1.78) | **<0.001** | 2.78(2.52-3.10) | 1.66(1.50-2.67) | **0.003** |
|  | TE (a.u.) | 0.72(0.65-0.77) | 0.79(0.73-0.82) | **<0.001** | 0.57(0.51-0.64) | 0.61(0.55-0.65) | **0.001** |

Data are presented as median, (quartile Q1-Q3). Statistical analysis was performed using paired Wilcoxon signed-rank tests. Abbreviations: MV, mechanical ventilation; SB_nose_, spontaneous nasal breathing; SE, Power-law spectral exponent (β); TE, Trajectory Entropy. a.u, arbitrary unit.

## Table S5. Data summary of different conditions

| **Regions** | **Ventral** | | | **Dorsal** | | |
| --- | --- | --- | --- | --- | --- | --- |
| **Group** | **MV+propofol** | **Propofol only** | **P-value** | **MV+propofol** | **Propofol only** | **P-value** |
| PSD (lower band) | 43.22 (39.75-59.69) | 33.89 (20.11-41.32) | **0.007** | 42.18 (20.53-58.27) | 19.11 (12.83-40.72) | 0.053 |
| SE (a.u.) | 2.69 (2.22-3.20) | 1.78 (1.59-2.32) | **0.007** | 2.27 (2.03-2.60) | 2.05 (1.57-2.28) | 0.162 |
| TE (a.u.) | 0.57 (0.48-0.62) | 0.65 (0.59-0.72) | **0.016** | 0.57 (0.51-0.64) | 0.64 (0.56-0.71) | **0.026** |

Data are presented as median, (quartile Q1-Q3). Statistical analysis was performed using non-parametric alternatives (Kruskal–Wallis or Mann–Whitney U tests) as appropriate. Abbreviations: MV, mechanical ventilation; SE, Power-law spectral exponent (β); TE, Trajectory Entropy
